# Weather Extreme Events, Farm Management, and Cocoa Yield in Ecuador

**DOI:** 10.3390/plants15192891

**Published:** 2026-09-22

**Authors:** Elena Piedra-Bonilla, Gabriel de Sampaio Morais, Joel Gómez, Heidy Toala, Laís Rosa-Oliveira, Nallely Molina-Velasco

**Affiliations:** 1 Facultad de Ciencias Económicas y Empresariales, Universidad Ecotec, Km. 13.5 Vía Samborondón, Samborondón 092302, Ecuador; 2 Empresa Brasileira de Pesquisa Agropecuária, Embrapa Mandioca e Fruticultura, Rua da Embrapa, s/n, Cruz das Almas 44380-000, BA, Brazil; gabriel.morais@embrapa.br; 3 Programa de Pós-Graduação em Economia, Universidade Federal da Integração Latino-Americana, Foz do Iguaçu 85870-650, PR, Brazil; 4 Mapfre, Torre Atlas, Calle 13A NO y Justino Cornejo, Guayaquil 090512, Ecuador; jgomez@mapfre.com.ec; 5 Secretaría Nacional de Gestión de Riesgos, Coordinación Zonal 8/5, Km. 0.5 Av. Samborondón, Samborondón 092301, Ecuador; heidy.toala@gestionderiesgos.gob.ec; 6 Departamento de Engenharia Agrícola, Universidade Federal de Viçosa, Campus Universitário, Viçosa 36570-900, MG, Brazil; lais.rosa@ufv.br; 7 Departamento de Ciencias Sociales y Humanidades, Universidad Autónoma Metropolitana, Unidad Cuajimalpa, Av. Vasco de Quiroga 4871, Ciudad de México C.P. 05348, Mexico; nmolina@cua.uam.mx

**Keywords:** *Theobroma cacao*, climate extremes indices, very heavy precipitation days, management intensity, Poisson pseudo-maximum likelihood

## Abstract

The frequency and intensity of extreme weather events are increasing, yet their implications for perennial tree crops such as cocoa remain understudied. Existing cocoa-climate evidence is based on mean climate variables, discrete shocks, and concentrated in West and Central Africa. This study addresses these gaps with plot-level cocoa evidence for Ecuador, the world’s second-largest exporter, linking the 2016 ESPAC survey to parish-level ET-SCI indices. We estimated the associations of three climate extreme indices: consecutive dry days (CDD), very heavy precipitation days (R20mm), and warm days (TX90p), on observed yield. Each index is summarized over 1-, 5-, and 10-year look-back windows to separate recent from cumulative exposure, within cross-sectional Poisson pseudo-maximum-likelihood (PPML) models with province fixed effects, survey weights, parish-clustered standard errors, and farm-level controls including management intensity. CDD showed no significant association, and TX90p estimates were imprecise, whereas R20mm was negatively associated with yield across all windows, with stronger evidence for longer windows. Each additional very heavy precipitation day (in the window average) was associated with 0.5–0.8% lower yield. Adjustment for management intensity attenuated this association in the intermediate window, although the cross-sectional design precludes a causal buffering interpretation. Recurrent very heavy precipitation emerges as a climatic risk for Ecuadorian cocoa.

## 1. Introduction

Climate change is altering not only mean temperature and precipitation but also the frequency and intensity of climate extremes [1]. These extremes are increasingly recognized as important pathways through which a changing climate affects crop yields [2,3,4]. A large body of evidence has quantified these effects for major annual crops such as maize, wheat, rice and sorghum, typically using panel data with year-to-year weather variation to isolate the impact of transitory shocks [5,6,7,8]. Perennial tree crops have received comparatively little attention, despite covering a large share of tropical agricultural land and supporting the livelihoods of millions of smallholder households [9,10].

Cocoa (*Theobroma cacao*) is a case in point. It is an economically important crop in many tropical countries, and its production is expected to be reshaped by climate change [11,12,13]. However, much of the existing cocoa–climate evidence is either process-based, simulating potential yield under future climate scenarios rather than estimating responses based on observed yields [14,15,16], or relies on correlations between production and mean climate variables, which may mask the role of extremes [17]. Studies that empirically examine climatic shocks have generally focused on discrete events, such as a single drought year, or on the potential buffering role of certification and farm management, rather than on continuous indices of climate extremes [18,19,20]. Moreover, the cocoa–climate literature is heavily concentrated in West and Central Africa, leaving cocoa-producing regions of Latin America, including Ecuador, one of the world’s leading producers of fine-flavor cocoa, comparatively underexplored.

This study addresses these gaps by estimating the associations of three climate extreme indices, consecutive dry days (CDD), very heavy precipitation days (R20mm), and warm days (TX90p), with cocoa yield across Ecuador’s cocoa-producing provinces. Three design choices distinguish the analysis. First, rather than relying on mean climate variables, we use indices from the Expert Team on Sector-Specific Climate Indices (ET-SCI)/ClimPACT2 framework [21], which are designed to characterize different dimensions of climate extremes and have been widely used in crop–climate studies but have been sparsely applied to a perennial tree crop. Second, each index is summarized over three exposure look-back windows ending in 2016, allowing comparisons of associations with cocoa yield across recent and multi-year climatic exposure periods. Third, the analysis draws on plot-level data from Ecuador’s 2016 Continuous Survey of Agricultural Surface and Production (ESPAC) [22], which allows control for farm-level characteristics typically unobserved in more aggregated studies, including cocoa type, plantation age, cropping system, and management intensity.

By combining these design choices, this study asks whether the three climate extreme indices (CDD, R20mm, and TX90p) are associated with cocoa yield in Ecuador, the world’s second-largest exporter [23], whether these associations differ across 1-, 5-, and 10-year look-back windows, distinguishing recent from cumulative exposure; and whether farm management moderates them. Given the cross-sectional design, these questions are addressed in associational terms. To our knowledge, it is among the first analyses to apply ET-SCI extreme indices to a perennial tree crop such as cocoa in Latin America using observed, plot-level yields, thereby generating evidence for climate risk and adaptation planning for cocoa-producing regions in the region.

## 2. Literature Review

### 2.1. Ecuador’s Background

Cocoa plays a pivotal role in Ecuador’s economy, as it is the country’s second-largest non-oil export, the top crop in terms of cultivated area, and the livelihood of more than 400,000 small-scale producers. Cocoa beans are grown in 21 of Ecuador’s 24 provinces, covering an area of more than 600,000 hectares in 2025. As a result, the country leads fine-flavor cocoa production, accounting for 63% of the global market, and ranks as the top exporter in Latin America and the second-largest globally [23,24]. Over the past two decades, the high-yielding CCN-51 clone has expanded substantially alongside traditional fine-flavor plantations, creating a productivity-quality duality that shapes both the agronomy and the economics of the sector.

An estimated 137 companies export cocoa and related products. In 2022, Ecuadorian cocoa exports increased by 54%, driven in part by a decline in productivity in competing countries such as Côte d’Ivoire and Ghana, which suffered severe impacts from climate change [25]. While shrimp and bananas generate significant foreign exchange in the trade balance, cocoa stands out for its upward price trend since 2023 [24]. According to reports from the International Cocoa Organization [26], these events led to a decline in cocoa production that affected global supply and raised concerns within the chocolate industry [27].

In Ecuador, the cocoa sector’s vulnerability to climate change has also intensified in recent years due to events such as prolonged droughts, heavy rains, and extreme temperature fluctuations that affected the main producing regions on the coast and in the Amazon. Extreme events can reduce cocoa productivity because drought prevents complete fruit development, while very extreme rainfall diminishes flowering and fruit set [28]. This provides evidence of the impact that extreme weather events have on agricultural products, as they can significantly limit the production of crops that depend on favorable climatic conditions.

The country’s cocoa belt experiences a pronounced climatic gradient, from the drier southern coast to the wetter central and northern lowlands and the Andean foothills (Figure 1). It is periodically affected by El Niño-Southern Oscillation (ENSO) events that bring anomalous rainfall to the coast. The reference year of the ESPAC data, 2016, coincided with the strong 2015–2016 El Niño episode [29], which should be considered when interpreting the cross-sectional associations estimated in this study.

Agriculture in Ecuador accounts for approximately 7.5% to 9.6% of the country’s Gross Domestic Product (GDP). Families predominantly carry out cocoa production, and smallholder farmers (80%) generate exports of more than USD 3.35 billion in 2024 [24]. Changes in the frequency and seasonality of extreme events may directly affect crop cycles and water availability [1,11], affecting the economy of these family farmers.

Therefore, integrating climate risk management into territorial and production planning is essential, especially in areas where agriculture is the primary economic livelihood. This issue requires a multidimensional analysis that considers not only productive and technical aspects but also the economic, territorial, and public policy implications arising from the interaction between extreme weather and agriculture [30].

### 2.2. State of the Art

Extreme weather events such as heavy rains, prolonged heat waves, severe droughts, and temperature extremes have increased risks to agricultural productivity, adversely affecting rural incomes, food security, and the sector’s economic stability. Recent studies suggest heterogeneous impacts, with effects varying significantly depending on geographic region, predominant cropping systems, and local socioeconomic conditions [2,31,32].

Ricardian analyses account for farmers’ long-term adjustment to local climatic conditions through changes in crop choice, production systems, technology, and irrigation [33]. However, family farming is more vulnerable than large-scale systems, reflecting structural limitations in adaptive capacity [32].

International evidence confirms this vulnerability to climate extremes, including soybean production in Brazil [5], cereal systems in the Ganges River basin in India [6], and farms in Germany [3]. However, the magnitude and dominant climatic drivers of yield losses vary among regions, crops, and production systems, so findings for annual crops cannot be assumed to transfer to a perennial such as cocoa.

For cacao (*Theobroma cacao*), the literature has emphasized three climatic stressors, though unevenly. Drought and dry-season water deficit are the most studied: simulation models identify water deficit as the leading climatic constraint in West and Central Africa [14,16], and observations confirm it, as during the 2015–2016 El Niño drought that reduced the main harvest in Ghana [34] and caused high tree mortality and yield loss in agroforests in Bahia, Brazil [13]. High temperatures have also received attention, since warm extremes reduce photosynthetic rates and impair growth and reproduction [35]. In India, Kumar et al. [36] found a negative correlation between maximum temperature and cocoa yield. Excess rainfall, by contrast, has been comparatively under-examined, even though heavy rain promotes waterlogging and the humid conditions that favor major fungal diseases such as black pod and frosty pod rot [37,38]. It can also disrupt flowering and pollination; in Ecuador, heavy rains in 2015–2017 severely affected flowering and fruit development [28].

Existing studies document a multidimensional vulnerability of Ecuadorian cacao in current production areas. Research by Ceccarelli et al. [11], using species distribution modeling, reveals high vulnerability of both cultivated and wild cacao in low-lying coastal areas where 70% of national production is concentrated. Plasencia-Vázquez et al. [39] show how climate change scenarios may reshape the potential distributions of cacao and *Moniliophthora roreri*, linking climatic suitability, crop distribution, and disease risk. However, these approaches do not directly quantify how extreme weather relates to realized cocoa yields, a gap that limits a comprehensive understanding of climate risk for the national sector.

Agronomic management is increasingly examined as a moderator of these climatic risks. In Ghana, Bunn et al. [40] developed adaptation recommendations for cocoa after identifying a lack of documentation on the effects of extreme droughts, floods, and temperature variations, and further evidence on certification and adaptation practices suggests that more intensively managed farming, through irrigation, shade, disease control, and input use, can partly buffer the effects of climatic stress on cocoa livelihoods and yields [18,19,20,41]. Whether such management attenuates the yield response to specific climate extremes, however, remains largely unexplored, particularly outside West Africa.

Therefore, the effects of extreme weather on cocoa cultivation should be considered within a system linking environmental, economic, technological, and social dimensions [9,15,35]. Econometric models have been used to estimate relationships between climatic conditions and observed agricultural yields [8], whereas species-distribution and suitability models use climate and crop-occurrence data to identify areas with conditions suitable for cocoa cultivation rather than yield responses [12].

## 3. Results

### 3.1. Descriptive Statistics

Table 1 summarizes the estimation sample of 3052 cocoa plots across 155 parishes. Average yield is modest and right-skewed, consistent with a sector dominated by smallholdings alongside a smaller number of high-yielding estates. Fine-flavor cocoa accounts for 42.96% of plots, with the remainder planted with the high-yielding CCN51 clone. The climate extreme indices show substantial variation across parishes, which identifies their cross-sectional variation: consecutive dry days (CDD) range from roughly 7 to 165 across parishes in the ten-year window, and R20mm from 0 to 65. This dispersion matters for interpretation because it implies that any non-result reflects an absence of effect rather than a lack of identifying variation. Notably, the between-parish standard deviation of CDD increases with the averaging window, whereas that of TX90p decreases, a point we return to when interpreting the heat indices.

### 3.2. Adjusted Associations of Weather Extreme Events with Cocoa Yield

Table 2 reports the covariate-adjusted association of each extreme index with cocoa yield, estimated by PPML with province fixed effects and clustered standard errors (Equation (2)). Three patterns emerge.

First, drought shows no detectable association with yield. The CDD coefficient is indistinguishable from zero in all three windows (0.0011, 0.0003, and 0.0002; all *p* > 0.70). Because CDD exhibits ample cross-parish variation, this is an informative null: the design could have detected a sizeable association had one existed, and it did not. A plausible interpretation is that the perennial, deep-rooted cocoa tree, together with irrigation in the drier parts of the coastal belt, buffers transitory water deficits, so that variation in consecutive dry days is not strongly reflected in yield.

Second, and in contrast, very heavy precipitation is significantly negatively associated with yield. The R20mm coefficient is negative in all three windows (−0.0052, −0.0081 and −0.0077), significant at the 5% level for the five- and ten-year windows and at the 10% level for the one-year window, implying that each additional R20mm day (in the window average) is associated with roughly 0.5–0.8 percent lower yield. A 10-day increase in the window-average R20mm, modest relative to the range observed across parishes (roughly 3 to 60 days) (Table 1), therefore implies approximately a 5–8% lower yield. Statistical significance indicates that the association is unlikely to arise by chance, whereas it is this cumulative magnitude, rather than significance in itself, that conveys its agronomical relevance. The association is somewhat larger in magnitude for the five- and ten-year windows than for the one-year window, though we do not formally test the difference between coefficients. This pattern is consistent with mechanisms that plausibly link excess water to lower cocoa yield, like waterlogging and disease pressure such as frosty pod (*Moniliophthora roreri*) and black pod rot (*Phytophthora* spp.), although these channels are not directly tested here [42]. Figure 2 displays the covariate-adjusted coefficients for all three indices and windows, along with their confidence intervals; Section 4.3 examines the R20mm result in more detail.

Third, heat extremes show no significant association with yield. The TX90p coefficients are negative at the one- and five-year windows (−0.0289 and −0.0302) yet imprecisely estimated (*p* ≈ 0.26–0.33), and the ten-year coefficient is small and positive (0.0086, *p* > 0.85). Because TX90p is a percentile-based index whose cross-parish dispersion collapses at the ten-year window, these estimates are best read as inconclusive rather than as evidence of no association.

### 3.3. Very Heavy Precipitation and Farm Management

Figure 3 shows the spatial distribution of very heavy precipitation at the parish level alongside cocoa yield at the province level. The map is descriptive and does not condition on the covariates in Table 2. Still, the visual coincidence between the high-rainfall belt and the lowest-yield provinces is consistent with the results reported there. Table 3 and Figure 4 report how the R20mm coefficient changes as confounders and then management intensity are successively added.

Table 3 reports the coefficients central to this analysis; the full specification, including the background climate controls and the quadratic plantation-age term omitted here for readability, is reported in Appendix A Table A2. Moving from the climate-only specification (−0.0050, not significant) to the model with confounders (crop type, plantation age, cropping system, and province fixed effects), the R20mm coefficient (5-year window) is −0.0081 and significant at the 5% level under conventional clustered inference: the covariate-adjusted association corresponds to a yield reduction of about 0.8 percent per very heavy precipitation day. Additionally, adjusting for management intensity (0–3 count of irrigation, fertilization, and pesticide use) shrinks the coefficient to −0.0036, which is no longer significant (*p* = 0.44) (Figure 4).

The parish-clustered bootstrap (499 replications; Appendix A Table A3) shows the same qualitative pattern, though with weaker statistical evidence for the covariate-adjusted association than conventional inference: the covariate-adjusted association is −0.0081 (*p* = 0.080 under the bootstrap, versus *p* < 0.05 in Table 3), the association additionally adjusted for management is −0.0036 (*p* = 0.437), and the attenuation between the two is −0.0045, which is statistically significant (*p* = 0.003). In substantive terms, the association between very heavy precipitation and lower yield is weaker among more intensively managed farms. The bootstrap also returns a proportion-attenuated estimate of 0.557, but with a very wide confidence interval (−1.49 to 2.60); we therefore do not report this ratio as a precise point estimate, since it is unstable when the covariate-adjusted association is close to zero. The qualitative result, a statistically significant attenuation once management is accounted for, and an additionally-adjusted association indistinguishable from zero, is robust, and also holds when management intensity is constructed as the first principal component of the three practices rather than as a simple count. Because management is not randomly assigned, this attenuation is a conditional association and should not be interpreted as evidence that management causally buffers the impact of very heavy precipitation.

Two caveats apply. Management intensity is not randomly assigned: more intensively managed farms may differ in unobserved capital and know-how correlated with yield. Hence, the decomposition describes a conditional association rather than a strictly causal mechanism. And because the cross-section corresponds to the 2016 El Niño year, the rainfall signal is conditioned on an anomalous ENSO state.

The control variables are themselves informative. Fine-flavor cocoa is associated with lower yield than the high-yielding CCN51 (a coefficient of −0.7165 in the fully adjusted specification, corresponding to approximately 51% lower expected yield, *p* < 0.01), consistent with the well-documented productivity–quality trade-off between Ecuador’s traditional, quality-differentiated cocoa and the high-yielding clone that has displaced it across much of the coastal belt [11]. Higher management intensity is positively associated with yield (*p* < 0.01), and intercropped (associated) systems are associated with roughly 28% lower yield than monocultures (*p* < 0.01), consistent with the trade-off between the per-hectare productivity of intensified monocultures and the diversification benefits attributed to shaded systems in the literature [18,43]. Plantation age is not significantly associated with yield in this specification.

#### Robustness

Appendix A Table A1 reports the robustness of the R20mm coefficient across functional forms, to excluding parishes with imputed climate data, and to an alternative, more extreme rainfall threshold, for all three look-back windows.

The PPML and OLS-in-levels specifications agree closely: R20mm is negatively and significantly associated with yield at the 5% level or better in all three windows for both estimators. The log specification, by contrast, retains the same sign but loses statistical significance across all windows (*p* > 0.35). Because the log model necessarily excludes zero-yield plots, this loss of precision is consistent with our choice of PPML as the main specification, which retains these informative zeros rather than discarding them.

Restricting the estimation sample to the parishes with directly observed, non-imputed climate data (2633 plots) leaves the very heavy precipitation association intact: the covariate-adjusted association remains negative and significant in all three windows (−0.0058, −0.0089 and −0.0084, respectively), and is, if anything, marginally larger in magnitude than in the full sample (−0.0052, −0.0081 and −0.0077). This is consistent with interpolation-induced measurement error attenuating the coefficient toward zero in the full sample, and indicates that the imputed parishes are not driving the association. The additionally adjusted association (with management intensity included) follows the same pattern as in the full sample: negative but not statistically significant in any window (−0.0028, −0.0039 and −0.0036), closely matching the additionally adjusted association reported in Table 3 (−0.0036 at the five-year window).

Replacing R20mm with the more extreme R30mm threshold yields a negative and significant association in all three windows, larger in magnitude than the R20mm coefficients (−0.0099, *p* < 0.05, at one year; −0.0168, *p* < 0.01, at five years; and −0.0185, *p* < 0.05, at ten years), consistent with the interpretation that more intense rainfall, rather than rainfall in general, is what is most strongly associated with lower cocoa yield.

## 4. Discussion

This study estimated the association between climate extremes and cocoa yield across Ecuador’s cocoa-growing provinces, using look-back windows for three extreme indices in a cross-sectional PPML design using ESPAC 2016 microdata. The central result is asymmetric: extremes of temperature and drought show no detectable effect on yield, whereas heavy rainfall significantly reduces it, and this rainfall effect is largely mediated by farm management.

### 4.1. Mechanisms Related to Excess Rainfall and Reduced Cocoa Yield

Our analysis identifies a negative association between very heavy precipitation and yield but does not measure the underlying biological process. The channels discussed below, waterlogging, disease pressure, and impaired flowering and fruit set, are therefore offered as plausible, literature-based explanations rather than mechanisms tested in this study. Excess water and waterlogging impair root function and favor the fungal diseases that thrive under wet, humid conditions, most importantly frosty pod rot (*Moniliophthora roreri*) and black pod (*Phytophthora* spp.) [42], These are leading causes of yield loss in Ecuador and the wider Latin American region [37,38]. In summary, climate change is not only affecting the areas where cocoa is grown for physiological reasons, particularly in relation to changes in water regimes, but also affects the distribution of pests and diseases affecting this crop [44]. Beyond disease, excess rainfall disrupts reproduction. Cocoa flowering and fruit set are highly sensitive to water relations and pollination success: unpollinated cocoa flowers abscise within about a day, and flower abscission is under hormonal (abscisic acid and ethylene) control. Heavy rainfall and flooding can further disrupt the breeding habitat of the midge pollinators (*Forcipomyia* spp.) and cause widespread loss of existing flowers, thereby depressing fruit set [45,46]. In Ecuador, during 2015, 2016, and 2017, the extraordinary rains altered the historical averages in precipitation, solar brightness, and environmental temperature, causing severe impacts on flowering, the ears’ development, and the growth of the cacao trees, which, in some Ecuadorian coastal areas the precipitation exceeded more than 500% of the historical averages [28]. In the center of origin in the Colombian Amazon, small changes (1.0%) in extreme precipitation, in just 30 years, showed a decrease of suitable habitat by 70.2% of the habitat for wild cacao, and 80.8% in the Pacific region, which is the most important area of wild cacao endemism in the world [47]. 

### 4.2. Reversing the Water Constraint: A Regional Contrast

This finding adds nuance to the broader cocoa–climate literature. Process-based work for West and Central Africa identifies dry-season water deficit as the dominant climatic driver of cocoa yield [14]. Despite that, Koissy andN’Zuéa [20] confirmed the existence of long-run dynamics between climate conditions and cocoa yields (1961–2016) in Côte d’Ivoire, indicating that rainfall positively affected cocoa yield in the short run but not in the long run; a 1% increase in rainfall in the long run would lead to a decrease in cocoa yield by 3.87%. In randomly chosen farms in Bahia, Brazil, Gateau-Rey et al. [13] found that the 2015–16 severe El Niño, which caused an unprecedented drought in cocoa agroforests, led to high cocoa tree mortality (15%) and severely decreased cocoa yield (89%); the drought also increased the infection rate of the chronic fungal disease witches’ broom (*Moniliophthora perniciosa*). On the other hand, cocoa production in wet areas, such as the humid coast in Latin America, is also vulnerable to climate change-related excess water, which is also associated with soil nutrient leaching and fungal diseases [48]. In Ecuador’s humid coastal belt, and especially in the 2016 El Niño year, the binding constraint appears to be the opposite: excess rather than deficit water. The rapid recurrence of unusually heavy rainfall in some coastal regions is expected to threaten up to 60% of current cocoa-growing areas, leading cocoa cultivation to migrate to higher-altitude areas away from the coast [10]. The relevant dimension of water stress therefore depends on the regional climate regime, which helps explain why our drought index is flat while the very heavy precipitation index is consistently negative. Earlier empirical work relating cocoa output to mean rainfall has also found weak associations [17]. By using extreme indices rather than averages, we recover a precipitation signal that means tend to mask.

### 4.3. Informative Nulls for Drought and Heat

The absence of a detectable drought or heat effect is itself informative, because the climate indices vary widely across parishes: the design had ample power to detect a sizeable effect and did not. Two interpretations are consistent with the agronomy. First, cocoa operates near its thermal optimum, which limits the scope for heat damage [14]. Second, the perennial, deep-rooted tree, together with irrigation in drier areas and agroforestry shade, buffers transitory water deficits. This reading is consistent with evidence that management and system characteristics, more than exposure alone, shape cocoa’s resilience to climatic shocks [18]. For heat specifically, the percentile-based index loses cross-parish dispersion at the longest window, so the heat result is best treated as inconclusive rather than evidence of no effect, and it would benefit from absolute-threshold indices in future work. By analyzing joint effects, a loss in the suitability of the Brazilian Amazon for cocoa planting by 2050 is likely, owing to decreased precipitation and increased temperature caused by climate change [49]. Opposite findings were estimated for Indonesia, where temperature was positively associated with average cocoa production, meaning that a 1 °C/month increase would increase average cocoa productivity by 5.41 ton/ha/month [50].

### 4.4. The Role of Farm Management

The very heavy precipitation coefficient is smaller in magnitude and no longer statistically significant once farm input-use intensity is additionally adjusted for. We describe this as an attenuation of the climate-yield association after accounting for management intensity. It is consistent with a buffering role of management but, given the observational design, does not demonstrate one. This suggests that intensive management plays a buffering role. It aligns with Thompson et al. [18], who find that farm management and sub-national context, rather than certification as such, drive smallholder resilience to a drought shock in Ghana. On the other hand, Amoako et al. [41] found that cocoa farmers in Ghana, who adopted a moderate or high number of adaptation strategies, experienced statistically significant improvements in income per hectare (about 5–12%) and food consumption scores (about 11–15%) compared with those who adopted few strategies. Interventions to promote adaptation to climate change will be required in cacao cultivation areas in Ecuador expected to be affected, including the use of tolerant genotypes [11]. Moreover, Díaz-Montenegro et al. [51] revealed that Ecuadorian cocoa farmers focus more on mitigating severe perceived impacts, such as price drops and production losses due to adverse weather, than on probability-based strategies. 

The pattern in our controls is also consistent with a productivity-resilience trade-off: the high-yielding CCN51 clone and monoculture are associated with higher yields, whereas the traditional Fine-flavor variety and shaded, intercropped systems yield less per hectare but are associated with quality differentiation. Because management is not randomly assigned, this attenuation should be read as a conditional association; intensively managed farms may differ in unobserved capital and know-how, rather than as a strictly causal mechanism.

### 4.5. Implications for the Crop-Specific Climate Literature

Our results contrast with the annual-crop literature, where drought and floods are typically the dominant yield-reducing extremes for maize, rice and sorghum [6,7]. The perennial, shade-adapted cocoa tree responds differently: short-run drought and heat are buffered, while cumulative excess rainfall, operating through disease and reproductive failure, emerges as the salient threat. This crop-specific pattern cautions against extrapolating annual-crop findings to perennials. It suggests that adaptation priorities for Ecuadorian cocoa under wetter extremes lie in drainage, disease management, and water management, complemented by management intensification, rather than in drought mitigation. Cocoa agroforestry management in Bolivia was shown to reduce climate change impacts and disasters in the social-ecological system of cocoa-based livelihoods, offering an alternative opportunity for Ecuadorian cocoa farmers [43]. Moreover, sustainable certification has been posited as a key governance mechanism to enhance the climate resilience of smallholder farmers, with a strong effect on the adoption of basic management. Thus, certification has some potential to enhance climate resilience [18].

### 4.6. Limitations

Four limitations are noted. Between-parish variation is ample for CDD and R20mm; for the percentile-based TX90p it falls sharply at the ten-year window, so non-results for that index and window are treated as inconclusive rather than as evidence of no effect. Appendix A Figure A1 maps the additional indices (CDD, R30mm, TX90p) at the parish level, confirming substantial cross-parish variation in each and supporting the informative-null interpretation of the drought and heat results reported in Section 4.2.

Second, the 2016 cross-section coincides with a strong El Niño [29], so the climate signal is conditioned on an anomalous ENSO state. Third, farm management intensity is not randomly assigned, so the mediation decomposition describes a conditional association rather than a strictly causal mechanism. Fourth, the survey’s record of crop losses, dominated by drought and pests, does not permit a strong external validation of the excess-water channel.

## 5. Materials and Methods

This section describes the empirical approach used to estimate the associations between climate extreme indices and cocoa yield in Ecuador. Section 3.1 defines the dependent variable and the covariates included in the analysis. Section 3.2 presents the empirical strategy, including the econometric specification, estimator, and identification strategy. Section 3.3 describes the data sources, including the ESPAC survey design and the construction of the climate extreme indices.

### 5.1. Variables

The dependent variable is cocoa yield, defined as production in metric tons divided by productive-age area (in hectares). Using productive-age area rather than harvested area in the denominator preserves plots with productive trees but no harvest as informative zeros, plausibly the plots most affected by an extreme event, rather than discarding them as undefined. Cocoa production begins around three years after planting and continues for up to 15 years [52].

We summarize extreme weather events using climate extreme indices computed with the ClimPACT2 (version 2; University of New South Wales, Sydney, NSW, Australia) , which implements the indices defined by the Expert Team on Sector-Specific Climate Indices (ET-SCI) [21]. Indices are constructed at the parish level and merged into each plot through its parish identifier. We retain three indices that capture distinct, physiologically relevant dimensions of climatic stress for cocoa: drought, excess water, and heat, because they are linked to abiotic stresses in agriculture [53], each acting on cocoa through a specific agronomic pathway [35]. CDD, the longest spell of days with precipitation below 1 mm, is a proxy for water deficit and the stress most emphasized in the West and Central African cocoa literature [14,35]. R20mm, the number of days with precipitation of at least 20 mm, is a proxy for excess water and associated disease pressure [37,38], a pathway documented for humid growing regions but comparatively under-examined [29]. TX90p, the percentage of days with maximum temperature above the local 90th percentile, is a proxy for heat, reflecting the temperature sensitivity of cocoa physiology [35,39]. Frost days were discarded because frost does not occur in lowland tropical cocoa areas. By selecting continuous ET-SCI indices for these three dimensions, we ground the analysis in the mechanisms most emphasized in the cocoa-climate literature while extending it to observed, plot-level yields. Each climate index is paired with a background control for the orthogonal climatic dimension, entered as a quadratic and matched to the same time window: rainfall indices (CDD, R20mm) are paired with mean temperature, and the heat index (TX90p) with accumulated precipitation. This helps separate the contribution of the extreme from the correlation between temperature and precipitation across parishes [8,54,55].

The choice of nested 1-, 5-, and 10-year look-back windows reflects the perennial biology of cocoa, whose yield in any given year depends not only on current-season weather but also on the physiological state accumulated over previous years through canopy and root reserves and the build-up of pests and diseases [53]. The w-year window comprises the reference year together with the w preceding years. The one-year window approximates a recent, transitory shock acting on the current flowering-to-harvest cycle. The five-year window captures medium-run cumulative exposure over which trees and farmers can partially adjust [56]. Ten-year windows summarize longer-run structural exposure spanning much of the tree’s productive life. Comparing the three horizons, rather than committing to a single window, allows the association between extreme and yield to be assessed across recent and accumulated exposure.

Farm-level controls comprise structural confounders and a management-intensity mediator. Among the confounders, crop type is captured by an indicator for Fine-flavor cocoa (the traditional, quality-differentiated variety) versus CCN51 (a high-yielding clone). We use this crop-type classification rather than the survey’s separate seed-origin variable (common, improved, national hybrid, international hybrid seed) because the two are empirically near-collinear—in this sample, 92% of CCN51 plots use improved or hybrid seed, and 96% of Fine-flavor plots use common seed—and crop type yields a more precise, agronomically direct confounder with a single, well-balanced (57%/43%) indicator, rather than four unevenly sized seed categories. Additional structural confounders are plantation age and its square and cropping system (monoculture versus intercropping); province fixed effects absorb time-invariant geographic and structural differences across the cocoa-growing belt.

Farm management, by contrast, is treated as an intermediate variable rather than a confounder: irrigation, fertilization, and plant-protection products are themselves plausible responses to climatic conditions, so adjusting for them changes the interpretation of the climate coefficient, from an association that reflects both direct and management-related pathways to one that holds management intensity constant (Section 3.2). We describe this as an intermediate-variable adjustment rather than causal mediation, given the cross-sectional design. Because adoption of these three practices is correlated at the farm level (pairwise correlations of 0.34–0.59, all *p* < 0.001), we do not enter them separately, as that would distribute their shared contribution ambiguously across collinear indicators. Instead, we construct a management-intensity index as the simple count of practices adopted (0–3: irrigation, fertilization, and plant-protection products). As a robustness check, we also constructed this index as the score of the first principal component of the three practices; the two versions are correlated at 0.9997 and yield virtually identical estimates of the R20mm coefficient, confirming that the equal weighting implicit in the count is not a meaningful simplification. Intercropping is excluded from this index and retained as a separate structural covariate, since it reflects a cropping-system choice that is empirically uncorrelated with the three input-based practices and conceptually distinct from input intensification.

### 5.2. Empirical Strategy

Identification is cross-sectional and rests on variation in climatic exposure, summarized over windows of different lengths, across parishes that have already adjusted to their local climate. In the spirit of the Ricardian approach [33], comparing places that differ in long-run climatic exposure characterizes associations net of the adjustments those places have already made, and the look-back windows probe how the association varies with the exposure window.

Climate is commonly treated as exogenous in economic analyses of agriculture, since farmers do not choose local climatic conditions and are plausibly unrelated to individual production decisions [54]. Nevertheless, in a cross-sectional design such as ours, long-run climatic exposure remains correlated across space with unobserved parish-level factors, like soil, altitude, drainage, disease pressure, and infrastructure, that may also affect yield. We therefore interpret our estimates as adjusted associations rather than causal effects, with province fixed effects absorbing time-invariant geographic heterogeneity at the provincial level.

Because yield is non-negative, right-skewed, and includes informative zeros, the effect of climate extremes is estimated by Poisson pseudo-maximum likelihood (PPML) following Correia et al. and Elofsson et al. [57,58]. PPML accommodates zeros naturally, is robust to the skewness that distorts least-squares estimates in levels, and yields coefficients interpretable as semi-elasticities. The model is estimated separately for each of the nine combinations of climate index and time window.

Yield is modeled as an exponential function of the covariates. For climate index *k* over window *w*, the baseline (raw) specification for plot *i* in parish *p* and province *j* is:

(1) $$E\left[\left.Y_{ipj}\right|\cdot \right]=exp\left(\beta_{0}+\beta_{0}X_{p}^{kw}+\gamma_{1}T_{p}+\gamma_{2}T_{p}^{2}\right),$$

where Y is yield, X is the climate extreme index (varying at the parish level), and T is the paired background control (mean temperature for rainfall indices, accumulated precipitation for the heat index) entered quadratically [8,54]. The coefficient on X is a semi-elasticity; the implied percentage change in yield per unit of the index is 100 × [exp(β) − 1], which for small coefficients is well approximated by 100 × β.

Adding the structural confounders and province fixed effects yields the covariate-adjusted association between the climate extreme and yield:

(2) $$E\left[\left.Y_{ipj}\right|\cdot \right]=exp\left(\beta_{0}+\beta_{0}X_{p}^{kw}+\gamma_{1}T_{p}+\gamma_{2}T_{p}^{2}+\delta F_{i}+\theta_{1}A_{i}+\theta_{2}A_{i}^{2}+\lambda C_{i}+\alpha_{j}\right),$$

where F is an indicator for Fine-flavor cocoa (vs. CCN51), A is plantation age (and its square), C is the cropping system (intercropping vs. monoculture), and α is the province fixed effect.

Because management practices are adaptive responses to climate rather than pre-existing farm characteristics, the management intensity index is introduced only in the third block, thereby isolating the direct effect of the weather extreme net of this adaptation channel. Additionally, adjusting for the management-intensity index gives:

(3) $$E\left[\left.Y_{ipj}\right|\cdot \right]=exp\left(\beta_{0}+\beta_{0}X_{p}^{kw}+\gamma_{1}T_{p}+\gamma_{2}T_{p}^{2}+\delta F_{i}+\theta_{1}A_{i}+\theta_{2}A_{i}^{2}+\lambda C_{i}+\alpha_{j}+\mu M_{i}\right),$$

where M is the management-intensity index, the change in the X coefficient after additionally adjusting for management (the difference between the two coefficients above) summarizes how much of the covariate-adjusted association is accounted for by farm management:

(4) $$Attenuation\;=\;\beta_{1}^{\left(2\right)}-\beta_{1}^{\left(3\right)},$$

with confidence intervals obtained from a parish-clustered bootstrap (499 replications). Given the cross-sectional design and the contemporaneous measurement of management, we interpret this difference as an attenuation under adjustment, not as a causally identified indirect effect. We report level and log specifications as robustness checks. Estimates are weighted by the survey expansion factors, and standard errors are clustered at the parish level, where the climate indices vary; with 155 parish clusters, cluster-robust inference is reliable. Province fixed effects are absorbed using the high-dimensional PPML estimator (ppmlhdfe) [57].

### 5.3. Data Sources

Farm-level data come from the 2016 Continuous Survey of Agricultural Surface and Production (ESPAC), conducted by Ecuador’s National Institute of Statistics and Censuses [22]. The ESPAC is an annual survey based on multiple-frame sampling that combines an area frame, in which the sampling units are territorial segments, with a list frame of the largest producers of selected crops, including a dedicated cocoa stratum. The survey covers the 23 provinces of continental Ecuador and is designed to be representative at the provincial level. We use the plot-level microdata and restrict the sample to plots cultivated with cocoa, a permanent crop. After excluding plots without productive area, the estimation sample comprises 3052 cocoa plots distributed across 155 parishes and 16 provinces. All estimates use the final expansion factor, which combines the area and list frames, so that results are representative of the Ecuadorian cocoa-growing population.

Climate data were obtained from the global meteorological forcing dataset of the Terrestrial Hydrology Research Group (THRG), described by Sheffield et al. [43]. The dataset was constructed by combining global observation-based datasets with the National Centers for Environmental Prediction–National Center for Atmospheric Research (NCEP–NCAR) reanalysis. We used daily precipitation (mm day^−1^) and daily minimum and maximum air temperature (°C), provided in NetCDF format at a spatial resolution of 0.25° × 0.25°, corresponding to approximately 28 km × 28 km at the Equator, for the period 2006 to 2016. Using the Climate Data Operators (CDO), the gridded climate data were interpolated using the Largest Area Fraction Remapping method to the spatial grid of Ecuadorian parishes, also represented in NetCDF format. This procedure assigned climate values to the grid cells associated with each parish, allowing the daily values to be obtained.

The climate dataset did not provide valid indices for every parish in the official Instituto Nacional de Estadística y Censos (INEC) territorial framework (1036 parishes), owing to a combination of parishes absent from climate records and mismatches between the cartography underlying the climate dataset and the current parish framework. For all parishes lacking a valid index, values were imputed by inverse-distance weighting (IDW) [59] of the five nearest parishes with valid data, with weights inversely proportional to the distance between parish centroids; centroids were computed from the official INEC parish cartography projected to UTM zone 17S (EPSG:32717), and missing values were excluded from the weighted average. In the cocoa estimation sample, 20 of 155 parishes (12.9%) carried imputed index values, affecting 419 of 3052 plots (13.7%); no parish in the estimation sample lacked climate data after imputation. Because interpolation introduces measurement error in the climate regressors, which biases coefficients toward zero, the estimated associations, and the null results for CDD and TX90p in particular, are conservative in this respect. The very heavy precipitation R20mm estimate is robust to the exclusion of the 20 parishes (419 plots) with imputed climate data across all three windows (Appendix A Table A1, Section Robustness). The total estimate is, if anything, marginally larger when climate data are restricted to directly observed values, consistent with attenuation from interpolation-induced measurement error. More generally, the climate grid (28 km) is coarse relative to the parish and plot scale of production. So localized climatic variation, particularly in convective heavy rainfall, is smoothed out. With the interpolation to the parish framework and the IDW imputation, this mismatch between the spatial scale of the climate data and that of cocoa production adds measurement error that, as noted, attenuates the estimated association. The reported effects are therefore conservative, and higher-resolution or station-based precipitation products could sharpen the analysis in future research.

The IDW imputation was implemented in Python (version 3.13.5; Python Software Foundation, Wilmington, DE, USA); all subsequent estimation was conducted in Stata 19 BE (StataCorp LLC, College Station, TX, USA). The Stata and Python code used for data construction, spatial imputation of climate indices, PPML estimation, robustness checks, and the mediation bootstrap was developed with the assistance of a generative AI tool (see Acknowledgments for product details). The authors executed, inspected, and validated all code against the ESPAC 2016 microdata, and all reported coefficients, standard errors, and statistical inferences result from the authors’ own estimation runs.

## 6. Conclusions

To our knowledge, this study provides the first cross-sectional estimates of the associations between climate extreme indices and cocoa yield in Latin America, using plot-level microdata and exposure windows of different lengths. The central finding is that very heavy precipitation, rather than drought or heat, shows the most consistent negative association with cocoa yield in Ecuador’s coastal growing belt, and that this association is substantially weaker among more intensively managed farms. This contrasts with the dry-season water-deficit narrative that dominates the West African literature, suggesting that the relevant dimension of climatic risk for cocoa may be regime-specific rather than universal.

Because the design is observational, these results point to potential adaptation implications rather than tested prescriptions. They suggest that, in humid coastal belts, measures addressing excess water during high-rainfall years, such as drainage and disease management, could be as relevant as drought-focused strategies. The association between more intensive input use and a weaker rainfall–yield relationship is consistent with a buffering role of farm management but does not establish causality. The productivity–quality trade-off between the CCN51 clone and the traditional Fine-flavor variety suggests that adaptation strategies may need to be tailored to crop type. Future work using a multi-year panel of ESPAC rounds, within-parish variation, and absolute-threshold heat indices would test whether these associations persist beyond a single El Niño year and whether the apparent buffering reflects long-run adaptation.

## Figures and Tables

**Figure 1:**
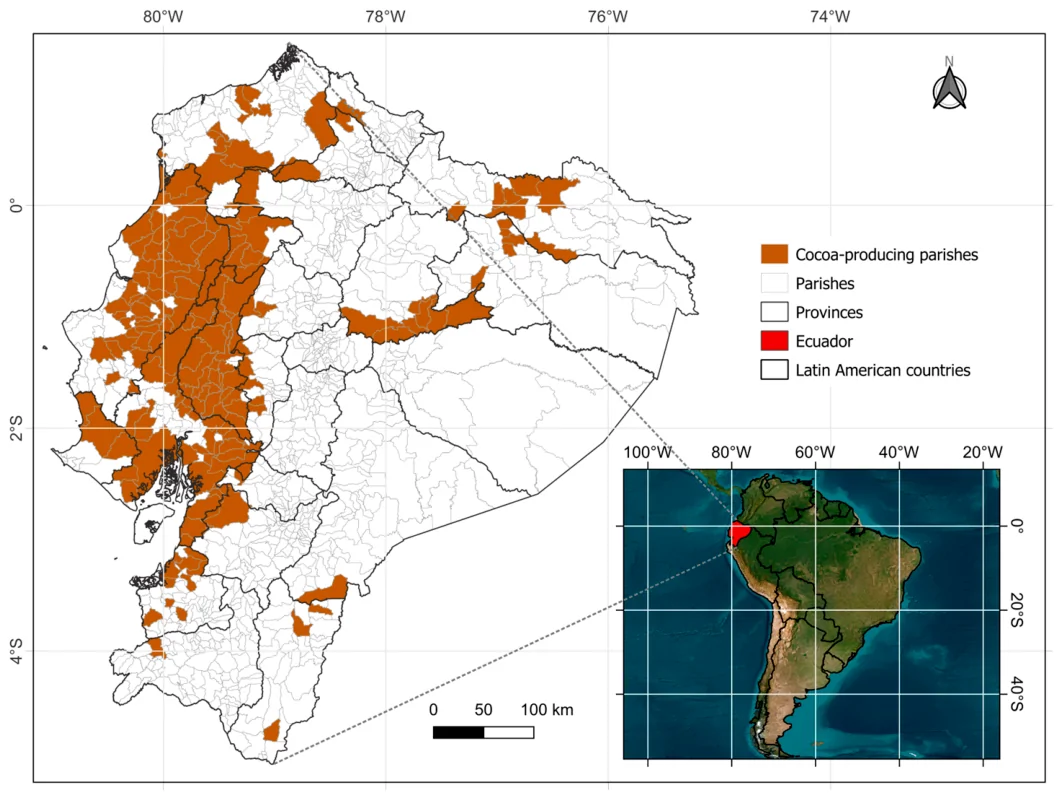
Study area showing cocoa-producing parishes across Ecuador in 2016. Administrative boundaries and cocoa-producing parishes: Instituto Nacional de Estadística y Censos (INEC) [22]. Maps prepared using QGIS 3.44.

**Figure 2:**
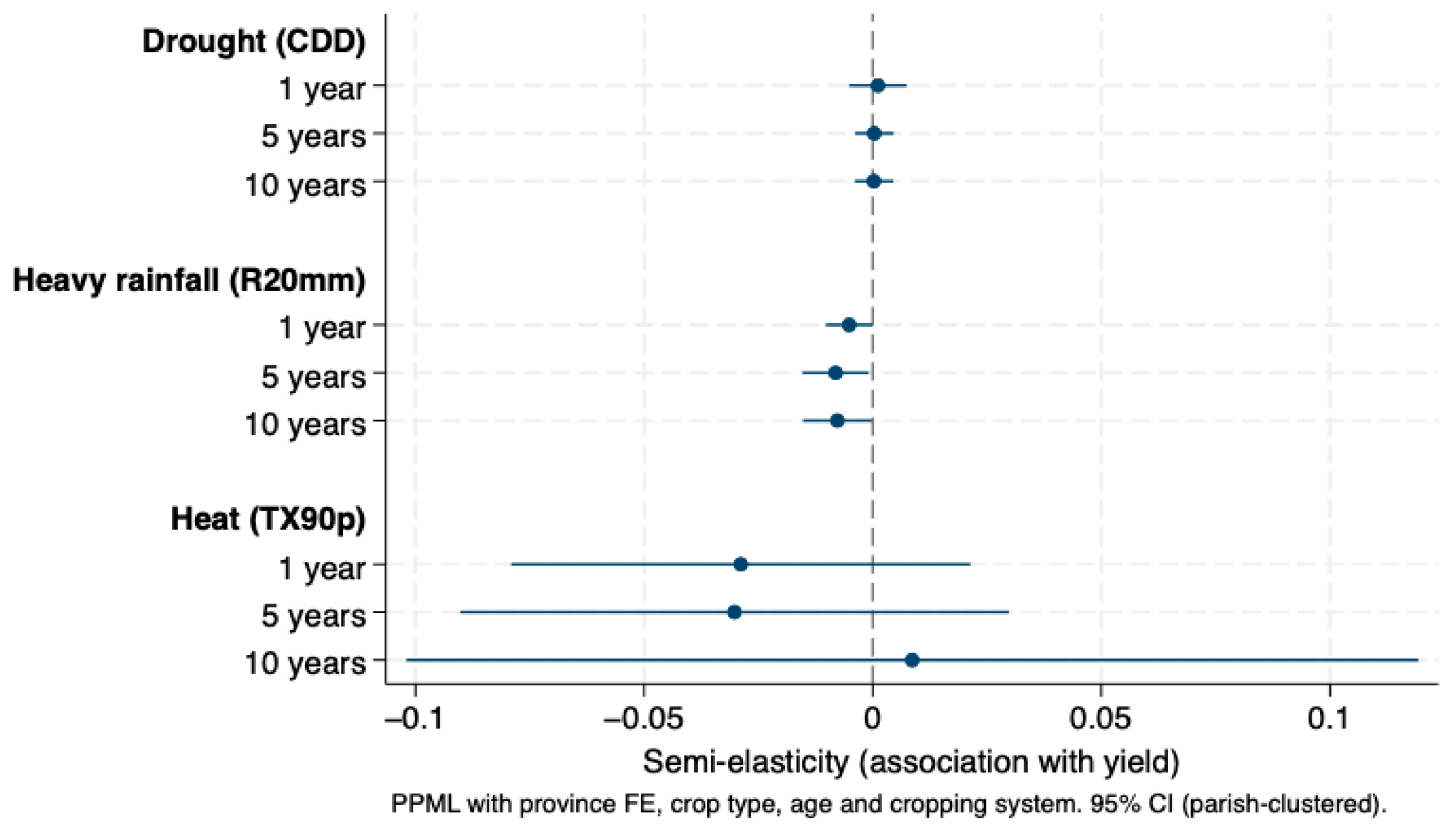
Adjusted association (semi-elasticity) of extreme weather events with cocoa yield across 1-, 5-, and 10-year look-back windows. Points are PPML coefficients; bars are 95% confidence intervals (parish-clustered) using Stata19 BE.

**Figure 3:**
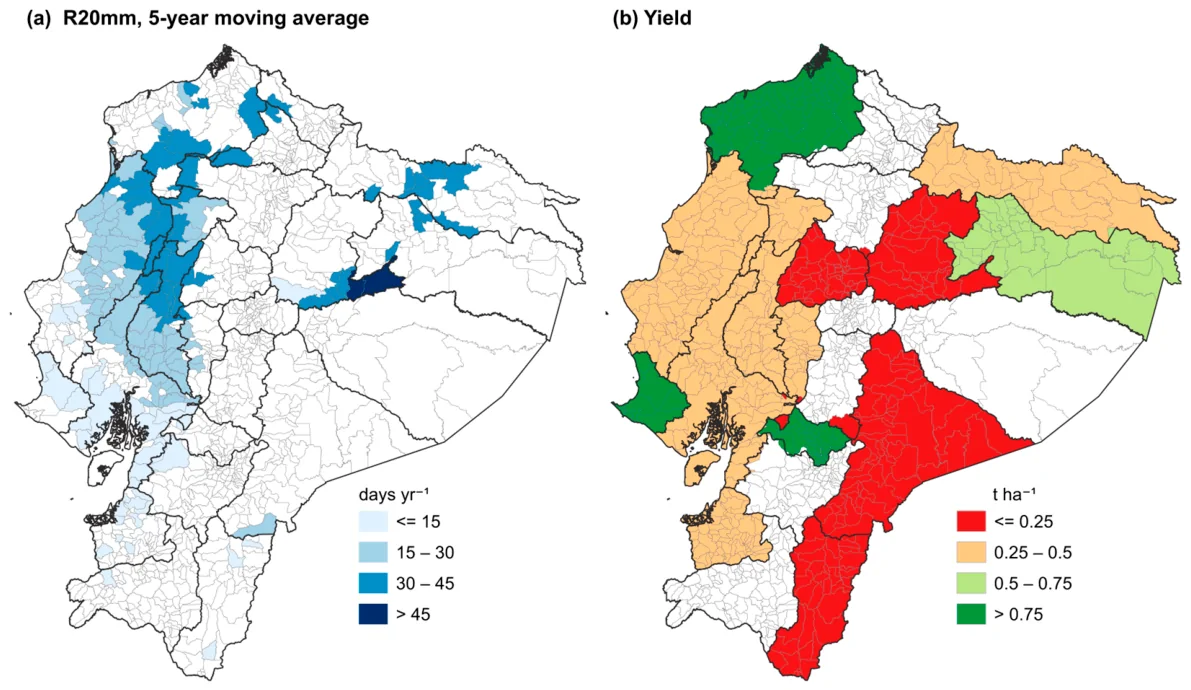
(**a**) Very heavy precipitation days (R20mm), 5-year look-back window, by parish. (**b**) Cocoa yield (t/ha), by province. Yield is mapped at the provincial level, the level at which ESPAC is representative; rainfall is mapped at the parish level, matching its use in the regressions using QGIS 3.44.

**Figure 4:**
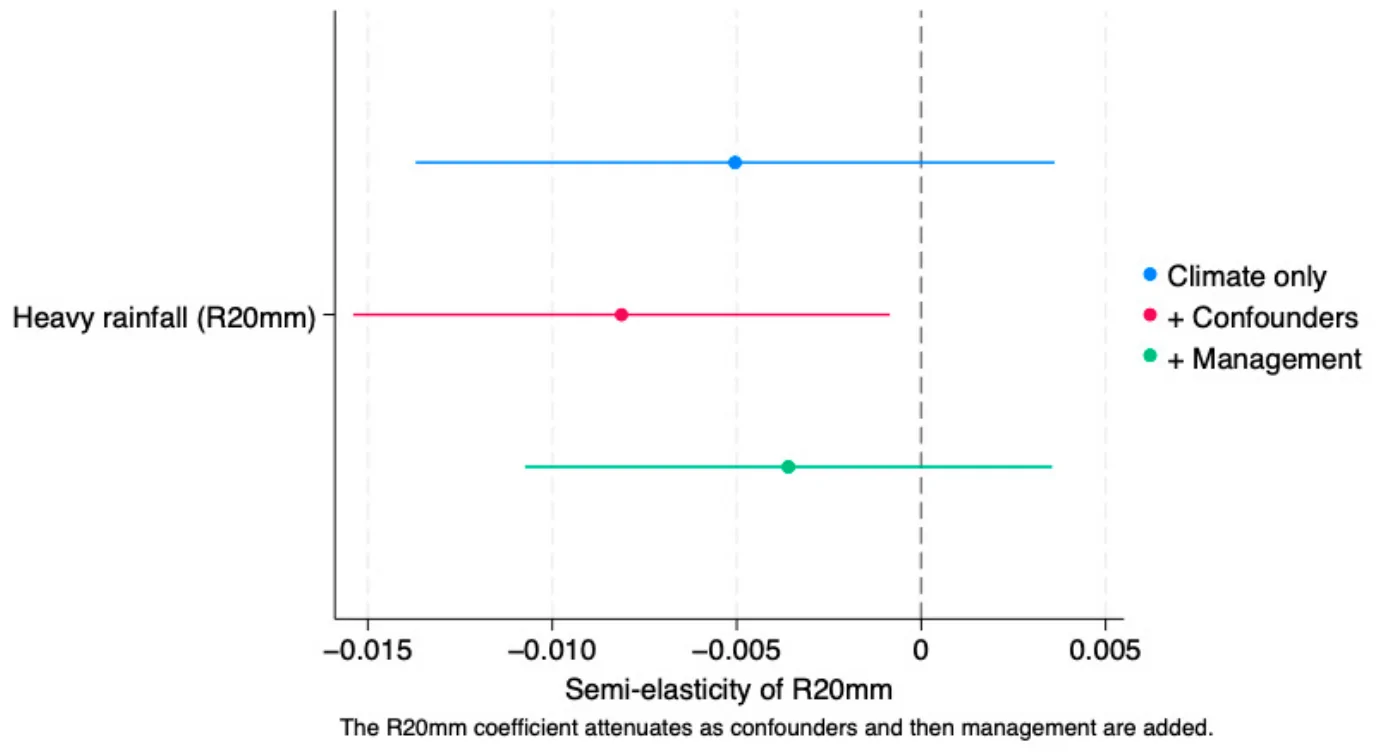
Very heavy precipitation (R20mm): attenuation under farm management intensity. 5-year window using Stata19 BE.

**Table 1:** Descriptive statistics for 1-, 5-, and 10-year look-back windows of climate extreme indices: consecutive dry days (CDD), very heavy precipitation days (R20mm), and warm days (TX90p).

Variables	Mean	SD	Min	Max
Yield (t/ha)	0.397	0.394	0.00	2.27
CDD, 1 y	29.109	15.656	6.50	114.03
CDD, 5 y	31.726	20.439	6.67	150.50
CDD, 10 y	30.018	20.909	7.00	165.09
R20mm, 1 y	26.816	13.170	2.50	51.00
R20mm, 5 y	27.786	9.522	2.83	56.17
R20mm, 10 y	26.909	9.699	3.55	61.18
TX90p, 1 y	15.954	1.868	0.40	18.88
TX90p, 5 y	14.234	1.743	0.13	16.97
TX90p, 10 y	10.495	0.950	3.95	12.32
Plantation age (years)	21.063	21.358	1.00	98.00
Fine-flavor (=1; vs. CCN51) *	0.479	0.500	0.00	1.00
Irrigation (=1)	0.249	0.433	0.00	1.00
Fertilizer (=1)	0.310	0.462	0.00	1.00
Pesticide use (=1)	0.362	0.481	0.00	1.00
Intercropping (=1)	0.104	0.306	0.00	1.00
Management intensity (0–3)	0.921	1.111	0.00	3.00

Note: Number of observations = 3052. Windows are labeled by look-back length; the w-year window spans the reference year plus the w preceding years. * Fine-flavor cocoa (the traditional, quality-differentiated variety) versus CCN51 (a high-yielding clone).

**Table 2:** Weather extreme events and cocoa yield: covariate-adjusted associations (PPML).

Extreme Indices	1 Year	5 Years	10 Years
Drought (CDD)	0.0011	0.0003	0.0002
	(0.0032)	(0.0021)	(0.0021)
Very heavy precipitation (R20mm)	−0.0052 *	−0.0081 **	−0.0077 **
	(0.0026)	(0.0037)	(0.0038)
Heat (TX90p)	−0.0289	−0.0302	0.0086
	(0.0256)	(0.0306)	(0.0565)
*N*	3052	3052	3052

Note: Standard errors clustered by parish in parentheses. Coefficients are semi-elasticities (×100 = %change per 1-unit change in the index). Windows are labeled by look-back length; the w-year window spans the reference year plus the w preceding years. Each model: PPML with province fixed effects, variety, age, and cropping system, and expansion factors using Stata 19 BE. * *p* < 0.10, ** *p* < 0.05.

**Table 3:** Very heavy precipitation and cocoa yield: adjusted associations.

Variables	(1)	(2)	(3)
	Climate Only	+Confounders	+Management
R20mm, 5 y	−0.0050	−0.0081 **	−0.0036
	(0.0044)	(0.0037)	(0.0036)
			
Management intensity (0–3)			0.1908 ***
			(0.0336)
			
Fine-flavor (vs. CCN51)		−0.8623 ***	−0.7165 ***
		(0.0958)	(0.0912)
			
Plantation age		0.0015	0.0047
		(0.0062)	(0.0057)
			
Intercropping		−0.3254 ***	−0.3311 ***
		(0.0702)	(0.0669)
*N*	3052	3052	3052

Source: Research results using Stata 19 BE. Standard errors clustered by parish in parentheses. ** *p* < 0.05, *** *p* < 0.01. For readability, this table omits the quadratic background-climate and plantation-age terms; the full estimation output is reported in Appendix A Table A2. Bootstrap (499 rep., 155 parish clusters): covariate-adjusted = −0.0081 (*p* = 0.080); additionally adjusted form management = −0.0036 (*p* = 0.437); attenuation after management adjustment = −0.0045 (*p* = 0.003). See Appendix A Table A3.

**Figure A1:**
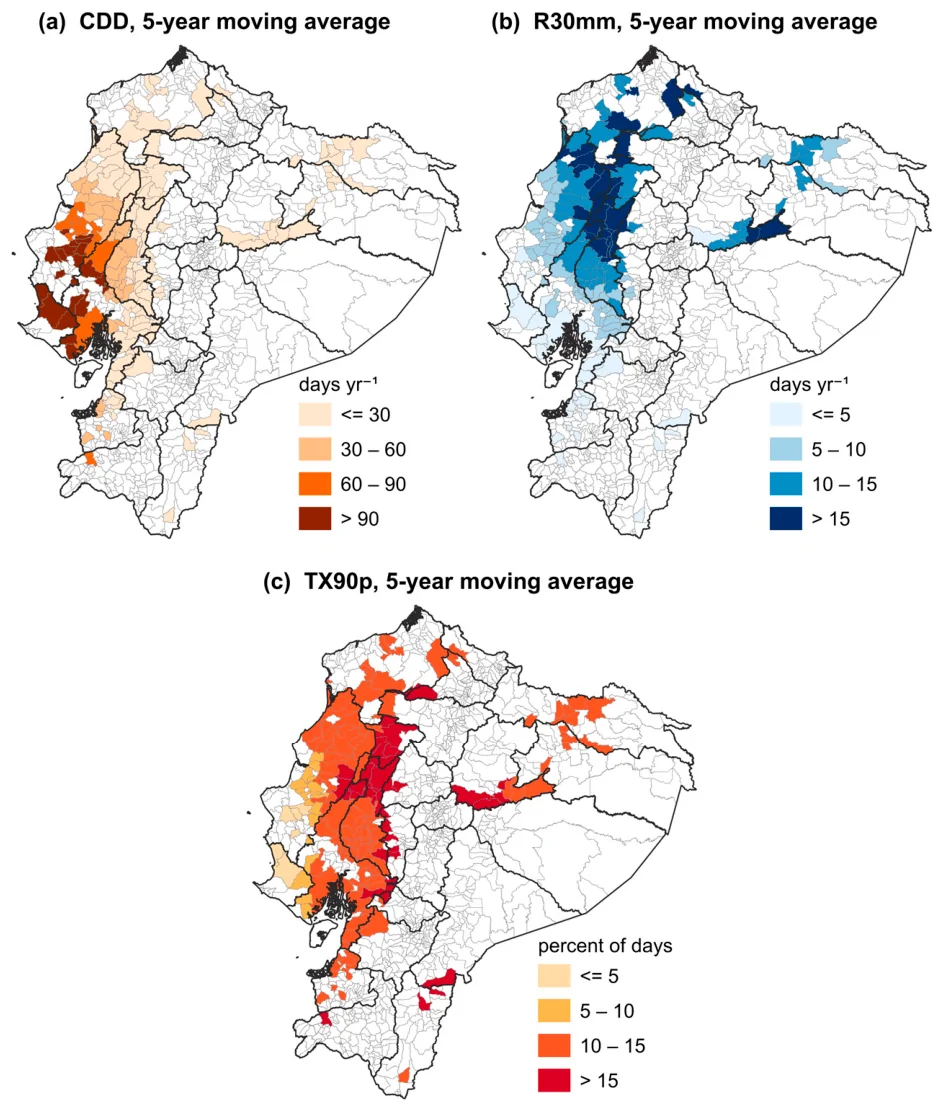
Spatial distribution of additional climate extreme indices at the parish level (5-year look-back window) using QGIS 3.44. (**a**) Consecutive dry days (CDD). (**b**) Very heavy precipitation (R30mm: Days with precipitation ≥ 30 mm). (**c**) Hot days (TX90p).

## Data Availability

The data and code supporting the analysis are available in Mendeley Data at https://data.mendeley.com/preview/z2vxr6j7rt?a=6d1be986-723d-4eaa-b556-5657c23768d5 (uploaded on 8 April 2026). The farm-level data presented in this study are available in Ecuador’s National Institute of Statistics and Production (INEC) at https://www.ecuadorencifras.gob.ec/encuesta-de-superficie-y-produccion-agropecuaria-continua-2015-2016-2017-2/ (accessed on 10 April 2025).

## Abbreviations

The following abbreviations are used in this manuscript:

ET-SCI	Expert Team on Sector-Specific Climate Indices
INEC	National Institute of Statistics and Censuses (in Spanish *Instituto Nacional de Estadística y Censos*) in Ecuador
ESPAC	Continuous Survey of Agricultural Surface and Production (in Spanish *Encuesta de Superficie y Producción Agropecuaria Continua*) in Ecuador
TX	Daily maximum temperature
TN	Daily minimum temperature
RR	Daily precipitation amount
CDD	Consecutive Dry Days (maximum number of consecutive days with RR < 1 mm)
R20mm	Number of very heavy rain days (Days with precipitation ≥ 20 mm)
R30mm	Number of very heavy rain days (Days with precipitation ≥ 30 mm)
TX90p	Amount of hot days (percentage of days when TX > 90th percentile)
THRG	Terrestrial Hydrology Research Group
NCEP—NCAR	National Center for Environmental Prediction/National Center for Atmospheric Research
IDW	Inverse distance weighting

## Appendix A

**Table A1:** Robustness of the very heavy precipitation R20mm and R30mm.

	(1)	(2)	(3)	(4)	(5)	(6)
	PPML	OLS (Levels)	Log	Excl. Imputed (+Conf.)	Excl. Imputed (+Manag.)	PPML, R30mm
1-year window						
R20mm	−0.0052 *	−0.0023 **	−0.0022	−0.0058 **	−0.0028	
	(0.0026)	(0.0010)	(0.0031)	(0.0027)	(0.0027)	
R30mm						−0.0099 **
						(0.0046)
5-year window						
R20mm	−0.0081 **	−0.0037 **	−0.0038	−0.0089 **	−0.0039	
	(0.0037)	(0.0014)	(0.0043)	(0.0038)	(0.0038)	
R30mm						−0.0168 ***
						(0.0064)
10-year window						
R20mm	−0.0077 **	−0.0035 **	−0.0034	−0.0084 **	−0.0036	
	(0.0038)	(0.0014)	(0.0044)	(0.0039)	(0.0039)	
R30mm						−0.0185 **
						(0.0076)
*N*	3052	3052	3052	2633	2633	3052
Parishes	155	155	155	135	135	155

Source: Research results using Stata 19 BE. Note: All models include mean temperature (quadratic), crop type (Fine-flavor vs. CCN51), plantation age (quadratic), cropping system (intercropping vs. monoculture), and province fixed effects, weighted by expansion factors, with standard errors clustered by parish in parentheses. PPML and log coefficients are semi-elasticities; the OLS (levels) coefficient is in t/ha per very heavy precipitation day. The log model excludes zero-yield plots. ‘Excl. imputed (+ Conf.)’ and ‘Excl. imputed (+ Manag.)’ restrict the sample to parishes with directly observed (non-imputed) climate data and report, respectively, the confounder-adjusted and the additionally-adjusted associations, mirroring the columns of Table 3. The last column replaces R20mm with R30mm. * *p* < 0.10, ** *p* < 0.05, *** *p* < 0.01.

**Table A2:** Full specification (companion to Table 3).

	(1)	(2)	(3)
	Climate Only	+Confounders	+Management
R20mm, 5 y	−0.0050	−0.0081 **	−0.0036
	(0.0044)	(0.0037)	(0.0036)
			
Mean temperature (5 y)	0.0609	0.0460	−0.0415
	(0.3734)	(0.4041)	(0.3867)
			
Mean temperature squared	−0.0014	−0.0011	0.0004
	(0.0077)	(0.0080)	(0.0077)
			
Fine-flavor (vs. CCN51)		−0.8623 ***	−0.7165 ***
		(0.0958)	(0.0912)
			
Plantation age		0.0015	0.0047
		(0.0062)	(0.0057)
			
Plantation age squared		−0.0001	−0.0001 *
		(0.0001)	(0.0001)
			
Intercropping		−0.3254 ***	−0.3311 ***
		(0.0702)	(0.0669)
			
Management intensity (0–3)			0.1908 ***
			(0.0336)
			
Constant	−1.4402	−0.7442	0.0888
	(4.4537)	(5.0758)	(4.8538)
			
Observations	3052	3052	3052

Note: This table reports all coefficients for the models summarized in Table 3, including the quadratic background climate and plantation-age terms omitted there for readability. Province fixed effects are included in the +Confounders and +Management models (16 provinces; coefficients not reported individually) using Stata 19 BE. Standard errors clustered by parish in parentheses. * *p* < 0.10, ** *p* < 0.05, *** *p* < 0.01.

**Table A3:** Attenuation of the R20mm association after adjustment for management (bootstrap).

Quantity	Coefficient	Bootstrap SE	*p*-Value	95% CI
Covariate-adjusted association	−0.0081	0.0046	0.080	[−0.0172, 0.0010]
Additionally adjusted for management	−0.0036	0.0046	0.437	[−0.0127, 0.0055]
Attenuation (difference)	−0.0045	0.0015	0.003	[−0.0075, −0.0015]
Proportion attenuated	0.557	1.044	0.593	[−1.488, 2.603]

Note: Bootstrap with 499 replications clustered by parish (155 clusters) using Stata 19 BE. The covariate-adjusted and additionally-adjusted associations correspond to the R20mm coefficient (5-year window) in the confounder-only and confounder-plus-management specifications reported in Table 3. The attenuation is the difference between the two. The proportion attenuated is reported for completeness; its confidence interval is wide because the covariate-adjusted association is close to zero and should not be read as a precise point estimate. CI = 95% bootstrap confidence interval.
